# Percutaneous direct pancreatic duct intervention in management of pancreatic fistulas: a primary treatment or temporizing therapy to prepare for elective surgery

**DOI:** 10.1186/s12876-021-01620-z

**Published:** 2021-01-28

**Authors:** Xi Li, Ricardo Paz-Fumagalli, Weiping Wang, Beau B. Toskich, John A. Stauffer, Gregory T. Frey, J. Mark McKinney, Justin H. Nguyen

**Affiliations:** 1 Interventional Radiology Department, The Fifth Affiliated Hospital of Sun Yat-Sen University, Zhuhai, Guandong China; 2 Division of Interventional Radiology, Mayo Clinic, Jacksonville, FL 32224 USA; 3 Department of General Surgery, Mayo Clinic, Jacksonville, FL USA; 4 Department of Transplantation, Mayo Clinic, Jacksonville, FL USA

**Keywords:** Pancreas, Postoperative complications, Stent, Suction drainage, Therapeutic embolization

## Abstract

**Background:**

This study evaluates preliminary results of image-guided percutaneous direct pancreatic duct intervention in the management of pancreatic fistula after surgery or pancreatitis when initially ineligible for surgical or endoscopic therapy.

**Methods:**

Between 2001 and 2018 the medical records of all patients that underwent percutaneous pancreatic duct intervention for radiographically confirmed pancreatic fistula initially ineligible for surgical or endoscopic repair were reviewed for demographics, clinical history, procedure details, adverse events, procedure related imaging and laboratory results, ability to directly catheterized the main pancreatic duct, and whether desired clinical objectives were met.

**Results:**

In 10 of 11patients (6 male and 5 female with mean age 60.5, range 39–89) percutaneous pancreatic duct cannulation was possible. The 10 duct interventions included direct ductal suction drainage in 7, percutaneous duct closure in 3 and stent placement in 1. Pancreatic fistulas closed in 7 of 10, 2 were temporized until elective surgery, and 1 palliated until death from malignancy. The single patient with failed duct cannulation resolved the fistula with prolonged catheter drainage of the peri-pancreatic cavity. There were no major adverse events related to intervention.

**Conclusion:**

In patients with pancreatic fistulas initially ineligible for endoscopic therapy or elective surgery, direct percutaneous pancreatic duct interventions are possible, can achieve improvement without major morbidity or mortality, and can improve and maintain the medical condition of patients in preparation for definitive surgery.

## Background

Pancreatic duct disruption can cause a leak of secretions and result in a pancreatic fistula, whether internal or external (if it reaches skin). Postoperative pancreatic fistula (POPF) or fistula caused by pancreatitis can be complicated by peri-pancreatic inflammation and infection, sepsis, hemorrhage, walled-off necrosis (WON) or disconnected pancreatic duct syndrome (DPDS). Pancreatic fistulas may need prolonged peri-pancreatic fluid collection (PFC) drainage and hospitalization, additional endoscopic or percutaneous procedures, or abdominal surgery. Management of pancreatic fistulas can be challenging in those with disruption of the main pancreatic duct or anastomotic fistula. Surgical management is often limited because of medical co-morbidities and increased perioperative morbi-mortality. Endoscopy is the first line therapy in managing pancreatic fistulas but its role may be limited in cases of DPDS or following gastroduodenal surgery [1]. While percutaneous drainage of PFC is widely utilized, a focal disruption of the duct can be an uncontrolled source of pancreatic secretions that can interfere with healing. Percutaneous interventions of the main pancreatic duct have been described but the experience reported is limited [2–4]. This study evaluates the feasibility, safety, and efficacy of percutaneous direct pancreatic duct interventions in the management of duct fistulas that were ineligible for, or had failed surgical and endoscopic management.

## Methods

### Patients

Institutional Review Board approval was obtained for this retrospective medical record review from 2001 to 2018. The review included all patients with clinically suspected pancreatic fistula who had fluoroscopic contrast evaluation of surgical or percutaneous drains. Fistulography in general was performed as previously described to aid in identification of the presence, location, and anatomy of the fistula [5]. Patients were included if the pancreatic duct fistula was confirmed by fluoroscopic observation of contrast opacification of the pancreatic duct, were not candidates for surgical or endoscopic management of the fistula, and then received a direct pancreatic duct intervention. Diagnosis of POPF was based on International Study Group of Pancreatic Surgery (ISGPS) criteria [6]. For purposes of uniformity in this report the ISGPS preferred term “fistula” will be adopted both for POPF and post-pancreatitis leaks, with the understanding that the ISGPS specifically addresses postoperative leaks and not leaks after pancreatitis.

Patients were then stratified into either postoperative or post-pancreatitis in etiology. Amylase assays were obtained from PFC drainage catheter fluid and were used to diagnose and guide treatment as has been previously published [6, 7], and to determine the time for drain removal. Demographics, clinical data, available imaging (CT scans, MRI, fluoroscopic studies, ERCP), procedural details, and outcomes were reviewed.

### Procedures

Percutaneous fluid collection catheter drainage (guided with computed tomography or fluoroscopy/sonography in an interventional angiographic suite) was required of all patients before attempting pancreatic duct cannulation. When needed a percutaneous necrosectomy (analogous to endoscopic necrosectomy) guided with fluoroscopy/sonography was also performed to evacuate the cavity debris (necrotic tissue, fibrinous conglomerates and/or saponified fat) using large bore drains through up-sized tracts up to 26 French, multiple saline washings, aspiration and evacuation of material with snares and with rigid forceps.

The access to the site of pancreatic fistula usually was through the existing percutaneous drain tract. If necessary a new access was created with either of two basic approaches. For non-anastomotic fistula (typically the terminal portion of duct after distal pancreatectomy or mid-duct after enucleation surgery or necrotizing pancreatitis) the PFC usually provided an anatomically useful approach but a separate new tract was created if needed to facilitate catheter manipulations. For anastomotic fistulas after pancreato-duodenectomy an enteric access was necessary to enable trans-anastomotic cannulation. This approach was similar to what an endoscopically placed trans-anastomotic stent would achieve, and was created with direct puncture of the jejunal loop, or through a trans-hepatic tract if a direct enteric access was not feasible [3, 4]. After the source of fistula was reached the duct was cannulated with angiographic catheters and guidewires. Typically a generic short tip angled 5 French catheter and a hydrophilic guidewire were steered into the duct, but coaxial 2.4–2.8 French microcatheters and 0.014″ guidewires were necessary for very small pancreatic duct openings or tight pancreatic anastomoses.

Once in the duct a contrast pancreatogram was obtained to illustrate the anatomy, which could include the entire duct, or show only a portion in cases of DPDS. In most cases, a multi-sidehole catheter (8.5F Dawson-Mueller drain, Cook Medical, Bloomington, IN) was positioned straight along the ductal lumen to establish external suction diversion of pancreatic juice. Catheter exchanges were performed to resolve catheter dislodgements, migration, or obstruction. The duct external drainage can be followed by placement stent (7 French, 7 cm, Geenen, Cook Medical Inc., Bloomington, IN) [2], if the physician determined that ductal continuity could be reestablished in cases of fistula along the main duct. For pancreato-jejunostomy anastomosis leak the cannulated duct was left to external drainage without other interventions. Distal pancreatectomy stump fistulas were treated with n-butyl cyanoacrylate (n-BCA, Trufill, Cordis Neurovascular Inc., Miami) and/or coil embolization (Tornado 0.018″ or 0.035 with 3–5 mm diameter, Cook Medical, Inc. Bloomington, IN) of the duct stump. To stop the production of pancreatic secretions in fistulas from DPDS the isolated duct was occluded with n-BCA, with additional occlusion with embolization coils at the interventionalist’s discretion. For fistulas originating elsewhere along the duct, if the patient’s condition was improved, and eligibility for surgery was regained, the ductal cannulation and drainage served to temporize until surgery. If a patient received a ductal cannulation to manage a postoperative leak, and later progressed to terminal cancer, the ductal external drainage unitl death was considered successful palliation of the leak.

Following ductal intervention, the patient was followed with imaging, laboratory and clinical findings and the final step was to remove all drains. A tube cavitygram was indicated when the PFC was resolved by CT scan or other imaging studies, the output was less than 15 cc per day, and the amylase level decreased to < 3 times the normal level [6]. The PFC drains were removed once the tube cavitygram confirmed absence of communication with the duct. Intraductal drains were left in place on continuous suction if the intent was to temporize until surgery. If surgery or other ductal interventions were not offered the ductal drain was removed after several weeks to determine if spontaneous closure of the fistula occurred. Follow up included a CT scan obtained at 3–6 months after completion of therapy.

### Outcomes analysis

The pancreatic duct interventions had different objectives depending on the clinical presentation. The critical technical goal was to achieve percutaneous catheterization of the pancreatic duct. The clinical objectives of the interventions included closure of pancreatic fistula, healing of pancreato-jejunal anastomotic or stump fistula, stabilization and improvement of the patient’s clinical condition as a temporizing measure until elective surgery, or control of a postoperative leak in a patient that developed terminal cancer after pancreatectomy. The drainage cavity fluid amylase levels and cavity size before and after completion of treatment with duct interventions were compared.

Adverse events were categorized according to Society of Interventional Radiology Clinical Practice Guidelines [8]. Post-procedural 30-day and overall mortality, and pancreatic fistula recurrence, were identified.

### Statistical analysis

Continuous variables were reported as mean (range), while categorical variables were reported as frequency (percentage). Continuous variables were compared by Mann–Whitney U test. All analyses were two-tailed and the threshold of significance was assessed at p < 0.05. The statistical analysis was performed using IBM SPSS Statistics, version 21, Chicago, IL.

## Results

### Patients

**Fig. 1:**
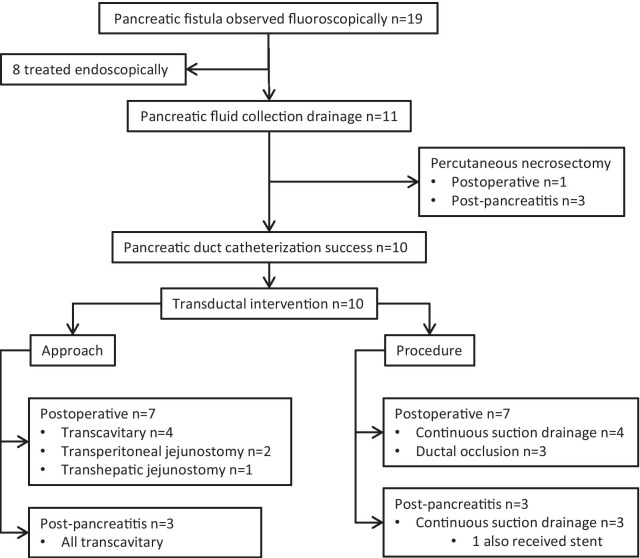
Procedural flowchart for postoperative and post-pancreatitis pancreatic duct fistulas that met inclusion criteria

A total of 11 patients (6 male and 5 female) received a percutaneous direct pancreatic duct intervention and were included for analysis (Fig. 1). The mean age was 60.5 (range 39–89). Of the 11 patients, 7 had postoperative fistula (all ISGPF grade B at baseline and 2 of 7 migrated to grade C when pancreatic surgery was performed) and 4 had post-pancreatitis fistula.

The mean duration of PFC drainage was 60.2 days (range 21–154) before pancreatic duct catheterization. The mean diameter of PFC was 4.0 cm (range 1–7) and the mean PFCs drain fluid amylase level was 36,465.5 U/L (range 1,213–134,000 U/L) at the time of catheterization. Percutaneous necrosectomy was performed in 4 of 11 patients. Of 11 patients, 8 were symptomatic with abdominal pain in 6, enlarging collection in 5; leukocytosis, fever and chills in 7, and 5 had nausea and vomiting. Three patients were asymptomatic and all had POPF. At baseline all 11 patients were considered poor surgical candidates, 4 of 11 were not eligible for endoscopic management, and 7 had failed endoscopy efforts. The clinical history, procedure details and clinical outcomes are presented in Table 1.

**Table 1:** Clinical, procedural and outcome details of pancreatic duct fistulas managed percutaneously

Patient	Diagnosis	Type of surgery	Presentation	Percutaneous procedures	Catheter drainage duration (days)	Outcome
1	Pancreatic neuroendocrine neoplasm	Partial resection/enucleation	Duct fistula at resection/enucleation site, DPDS (tail)	Percutaneous drain, percutaneous necrosectomy, disconnected duct catheterization through cavity, duct occlusion with n-BCA and coils	114	PFC resolved. Disconnected pancreas defunctionalized. Fistula closed
2	Pancreatic ductal adenocarcinoma	Pancreatico-duodenectomy	Anastomotic fistula	Surgical drain exchange, trans-peritoneal jejunostomy and duct catheterization for external diversion of secretions	65	PFC resolved. Fistula palliated with direct diversion of pancreatic secretions until death from liver metastases
3	Pancreatic ductal adenocarcinoma	Distal pancreatectomy and splenectomy	Stump fistula	Percutaneous drain, duct catheterization through cavity, and duct occlusion with n-BCA and coils	77	PFC resolved. Stump fistula closed
4	Pancreatic ductal adenocarcinoma	Pancreatico-duodenectomy	Anastomotic fistula	Surgical drain exchange, trans-peritoneal jejunostomy, and duct catheterization for complete external diversion of secretions	131	PFC resolved. Anastomosis healed and fistula closed
5	Chronic pancreatitis	Pancreatico-duodenectomy	Anastomotic fistula	Percutaneous drain, trans-hepatic jejunostomy, and duct catheterization for complete external diversion of secretions	66	PFC resolved. Anastomosis healed and fistula closed
6	Pancreatic ductal adenocarcinoma	Distal pancreatectomy and splenectomy	Stump fistula	Percutaneous drain, duct catheterization through cavity, and duct occlusion with coils	28	PFC resolved. Stump fistula closed
7	Renal cell carcinoma metastasis	Partial resection/enucleation	Duct fistula at resection/enucleation site, DPDS (tail)	Percutaneous drain, duct catheterization through cavity for complete external diversion of secretions	68	PFC resolved. Temporizing therapy until elective surgical pancreatico-jejunostomy
8	Acute necrotizing pancreatitis		WON, DPDS (body and tail)	Percutaneous drain, percutaneous necrosectomy, duct catheterization through cavity for complete diversion of secretions, and placement of internal pancreatic stent	168	PFC resolved. Disconnected duct continuity re-established with internal pancreatic duct stent. Ductal disruption healed
9	Acute necrotizing pancreatitis		WON, DPDS (tail)	Percutaneous drain, percutaneous necrosectomy, and duct catheterization through cavity for complete external diversion of secretions	154	PFC resolved. Temporizing therapy until elective surgical pancreatico-jejunostomy
10	Acute necrotizing pancreatitis		WON	Percutaneous drain, percutaneous necrosectomy, and duct catheterization through cavity for complete external diversion of secretions	181	PFC resolved. Ductal disruption healed
11	Acute necrotizing pancreatitis		DPDS (tail)	Percutaneous drain, percutaneous necrosectomy, failed duct catheterization through cavity	363	PFC resolved with prolonged drainage only. Ductal disruption healed

### Procedures performed

**Fig. 2:**
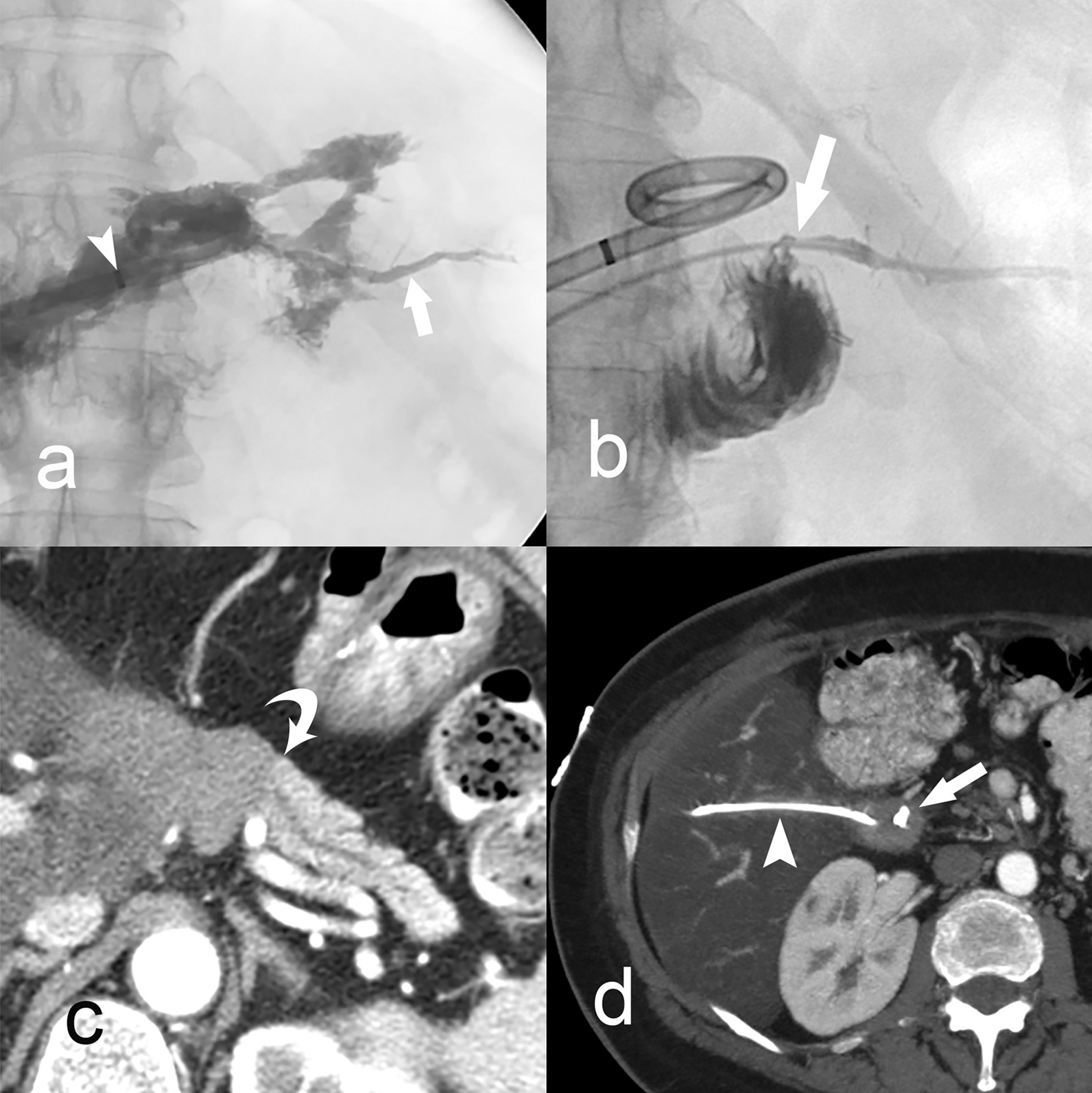
Patient 5 illustrates the technique of percutaneous Roux-en-Y jejunostomy to access the pancreatic duct for management of an anastomotic fistula after pancreato-duodenectomy. Contrast cavitygram (**a**) through the percutaneous drain (arrowhead) shows the pancreatic duct (arrow). CT scan image (**b**) shows the transhepatic trajectory of the percutaneous catheter (arrowhead) entering the Roux-en-Y afferent jejunal limb (arrow). After 2 months of continuous suction of pancreatic secretions through the percutaneous catheter contrast injection (**c**) shows that the anastomosis (arrow) is completely healed and the pancreatic duct normally drained into the jejunal limb. The catheter was removed. Follow up CT scan (**d**) 13 years later shows a normal pancreatojejunostomy (arrow) and all peri-pancreatic inflammation completely resolved

**Fig. 3:**
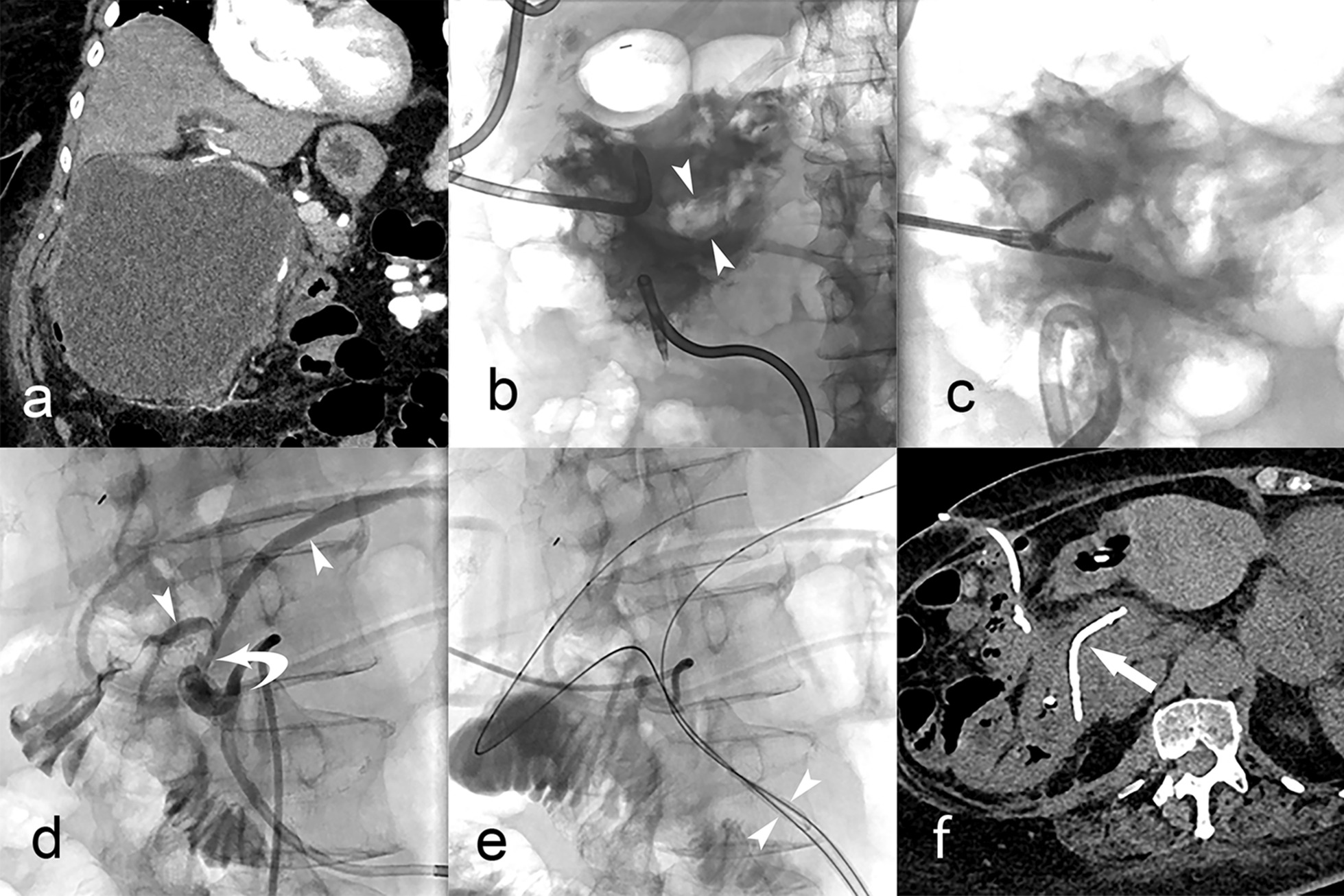
Pancreatic duct stump fistula closed with embolization coils. Patient 6 was found to have a distal pancreatectomy stump fistula that persisted despite endoscopic pancreatic duct stent placement. **a** Contrast injection into the surgically placed drain shows persistent pancreatic duct stump fistula. Notice contrast flowing along pancreatic duct and the endoscopic stent is faintly visible (arrow). **b** Pancreatogram after duct catheterization shows the percutaneous access sheath placed next to the non-healed duct stump (arrow). **c** The stump fistula was closed with embolization coils (arrow) without recurrence

**Fig. 4:**
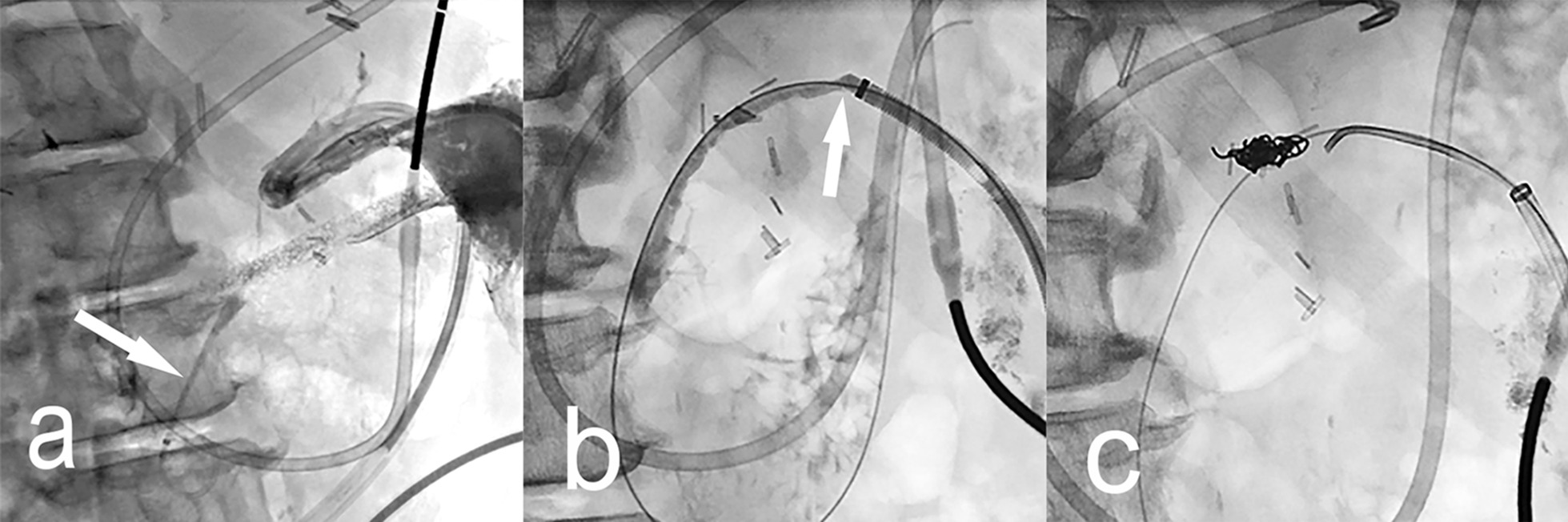
Disconnected pancreatic duct syndrome managed with transcavitary duct stent placement. Patient 8 presented with severe necrotizing pancreatitis. **a** Coronal reconstruction of abdominal CT scan shows a very large peri-pancreatic walled-off necrosis (WON). **b** Abdominal radiograph during contrast injection into the WON cavity 2 weeks of drainage shows improvement but persistence of amorphous filling defects caused by debris (arrowheads). **c** Radiograph during percutaneous necrosectomy shows a forceps introduced through a peel-away sheath grasping the large conglomerates of necrotic and fibrinous material during the process of evacuation. **d** After resolution of the peri-pancreatic WON a cavitygram shows communication with the pancreatic duct (arrowheads) through a focal disruption (curved arrow) at the neck of the pancreas. **e** Plastic stent in transit through the percutaneous tract is faintly visible as it is advanced in a folded configuration (arrowheads) over parallel guidewires, one directed towards the pancreatic tail and the other through the ampulla. **f** CT scan shows the plastic stent appropriately positioned in the pancreatic duct bridging the head and tail segments of pancreatic duct. After one month the stent was removed endoscopically and 6 months later the patient had no recurrence of peri-pancreatic abnormalities and no need for pancreatic enzyme supplementation

Percutaneous pancreatic duct cannulation was achieved with fluoroscopic guidance through the PFC cavity in 7 of 11 patients, through a percutaneous trans-peritoneal jejunostomy in 2, and through a percutaneous trans-hepatic jejunostomy in 1 (Fig. 2). Only 1 duct cannulation failed because a guidewire and catheter could not be inserted into the duct via trans-cavitary approach (Table 1, case #11). Therefore the technical success rate for pancreatic duct cannulation was 90.9% (10 of 11). After duct cannulation, subsequent direct ductal interventions in 10 patients, included external duct suction drainage with 8 French Dawson-Mueller multi-sidehole drains in 7 of 10 (anastomotic fistula in 3, WON in 3, and tumor enucleation site in 1), duct embolization in 3 of 10 (coils and n-BCA in one DPDS, coils and n-BCA for one duct stump fistula, and coils only in one duct stump fistula) (Fig. 3), and stent placement in 1 case of DPDS (Fig. 4). There were no coil migrations or non-target n-BCA embolization.

### Outcomes

Following direct pancreatic duct intervention, the PFC cavity resolved completely at a mean of 45.6 days (range 13–117). The mean body fluid amylase level decreased from 36,466 U/L (range 1,213–134,000) to 113 (range 22–354) before PFCs drain removal (p < 0.01). Fistulas resolved in 7 of 10 (70%) with mean duration of 38 days (range 7–117) from the time of duct catheterization to fistula closure. Two patients with DPDS were temporized until surgery at 47 and 96 days, and 1 patient died of pancreatic malignancy 25 days after placement of the ductal drain, before the POPF had closed, but had effective palliation of the peri-pancreatic inflammation caused by the pancreatic leak until death. No major adverse events or 30-day mortality were observed. Minor adverse events observed in 5 of 10 patients, including drain migrations and drain obstructions that required catheter exchanges. For the 10 patients in whom duct catheterization was achieved the interval from the date of initial PFC drainage to the date of removal of all catheters was 105 days (range 28–181). The single patient for whom the duct cannulation failed required a very prolonged PFC catheter drainage of 363 days until the fistula finally closed (Table 1). Mean follow-up time for the 11 patients was 37.4 months (range 1–108). There were no late recurrences of pancreatic fistula.

## Discussion

The case series presented herein shows that only 11 patients in a facility with a high volume of pancreatic disease received direct pancreatic duct percutaneous interventions in an 18-year period, underscoring the effectiveness of endoscopy, percutaneous catheter drainage, surgery or a combination of these well-established approaches [9–15]. Also, the great majority of patients with POPF and necrotizing pancreatitis were excluded because at no time during their management was the pancreatic duct seen radiologically. This analysis cannot estimate how often direct duct intervention can be done because it lacks precise total numbers of postoperative and post-pancreatitis patients during the 18-year period studied. We expect that the number of patients that benefit from pancreatic duct cannulation and interventions will grow with increasing awareness of the interventional techniques presented in this series.

Endoscopic and percutaneous drainage of PFC have been compared in the last 10 years. Overall, endoscopic and percutaneous drainage are similarly effective and complementary interventions for PFC [9, 16]. When direct pancreatic duct interventions are needed, endoscopy is the method of choice. Endoscopic stenting is most frequently performed, but sometimes it can be technically challenging or impossible, especially in patients with DPDS or following gastroduodenal surgery [1].

Pancreatic duct cannulation was done with one or more of the following objectives: diversion of pancreatic secretions, occlusion of a leaking duct, and/or pancreatic duct stent placement. The main reason for direct duct cannulation was to drain the pancreatic juice at the source and prevent enzymes from flowing into the peri-pancreatic space, with the intended effect of reducing the peri-pancreatic effusion, inflammation, accumulation of debris, and extent of the fistula, among other consequences. The decision for pancreatic duct percutaneous interventions was made after discussion with the surgical and gastroenterology physicians to determine the potential for repair, resection, or endoscopic treatment options. It was understood that endoscopic therapy is the therapy of choice, followed by surgery when appropriate. Therefore only a minority of patients deemed high-risk or ineligible for surgery or endoscopy, or who failed such therapy, were appropriate for percutaneous intervention.

In this group of patients the pancreatic duct cannulation was achieved in 10 of 11. When duct cannulation is possible, the desired clinical objectives can be met most patients. It can be argued that with sufficient time in many cases the fistula will heal with catheter drainage alone and direct duct intervention may be unnecessary. We certainly have observed this to occur but it may require a very long time, as illustrated by the single case of failed duct cannulation in this series that required 1 year of catheter drainage (twice as long as the longest drain dwell time in the group that received direct duct cannulation). Our experience shows that when traditional therapeutic maneuvers fail or are not possible, percutaneous pancreatic duct intervention substantially speeds the resolution of the fistula or provides temporization until surgery.

Diversion of the high output of pancreatic secretions effectively controlled enzymatic spillage to adjacent tissues. An important advantage of this method (in addition to diverting the pancreatic juice) is that the drain itself can also be used to monitor the healing process, identify when the fistula has closed, and determine the best time to remove the drain by doing contrast injections through the catheter. This methodology was used in all 10 patients with a direct ductal drain (2 temporized until surgery, 7 with ductal intervention as primary therapy, and 1 palliated with direct ductal drainage until death from cancer). Minimally-invasive duct occlusion techniques close pancreatic fistulas quickly and safely, preventing the need for surgery [17–19]. Duct occlusion can also simulate the effect of pancreatectomy of an isolated segment of gland [4]. In this instance occlusion/embolization renders the isolated pancreas non-functional and eliminates the fistula (patient #1), which would be the objective of surgical resection of the expendable portion of pancreas. Full preservation of the functional pancreas would be best achieved with a pancreato-jejunostomy or pancreato-gastrostomy. Interventional techniques can also enable placement of an internalized endoscopic-type plastic stent that bridges across both portions of the duct to re-establish continuity and secretion drainage into the duodenum (Fig. 3) [2]. We did not observe any ductal rupture or pancreatic secretion leaks provoked by duct occlusion with n-BCA or coils.

Because the study group is small and heterogeneous, comprising a collection of different clinical settings and procedures performed, it is speculative to draw broad clinical conclusions, but it does show that pancreatic duct interventions are potentially safe, with only minor adverse events and no procedure-related 30-day or delayed mortality in this series. Endocrine and exocrine function did not worsen as a result of the interventions. There were no identified cases of new onset or worsening diabetes or worsening of pancreatic enzyme deficiency in the cases of DPDS, probably because pancreatic secretion from the isolated segment had already stopped reaching the bowel lumen. Other complications including bleeding, sepsis and peritonitis were possible but not observed.

## Conclusion

Image-guided percutaneous pancreatic duct interventions were possible, achieved the desired clinical objectives in most patients without major morbidity or mortality, and improved and maintained the medical condition of patients in preparation for definitive surgery. This therapy can be considered for patients with pancreatic duct fistulas who are ineligible for surgery or have failed or do not qualify for endoscopic therapy.

## Data Availability

Data analyzed for this study are included in this article, and can be found in Table 1 and Figs. 1 through 4. The data generated by and used in the study is available from the corresponding author upon reasonable request.

## Abbreviations

POPF: Postoperative pancreatic fistula
WON: Walled-off necrosis
DPDS: Disconnected pancreatic duct syndrome
PFC: Peri-pancreatic fluid collection
ISGPS: International Study Group of Pancreatic Surgery
CT: Computed tomography
MRI: Magnetic resonance imaging
ERCP: Endoscopic retrograde cholangio-pancreatography
n-BCA: *N*-Butyl cyanoacrylate
